# Clinical Society

**Published:** 1897-11-20

**Authors:** 


### CLINICAL SOCIETY.

### Forcible Straightening in Potts' Curvature of the Spine.

There is something extremely fascinating in the idea of immediately reducing an angular curvature and at one blow removing a deformity which otherwise must leave the patient a hunchback for life, and when we are assured on competent authority that the thing has been done in a large number of cases and that accidents have been but few, the temptation is strong to go and do likewise, notwithstanding certain warnings given by the surgical instinct. We hope, however, that surgeons will not rush this matter, but will proceed with much caution until we know much more than we do at present in regard to the final results.

Putting on one side, however, all question of after consequences, it is a great fact that the thing can be done at all, and we hardly know which is the most surprising—the small amount of force which is in some cases required to bring the spine back to its position, or the fact that the patient escapes immediate and very serious injury from the large amount of force which in some cases is found necessary. One of the speakers at the Clinical Society last week said that the application of the plaster jacket was apt to be a somewhat ghastly sight. If, however, one seeks for the horrible, we must admit that the vision of six strong men pulling at one little child, and blindly forcing the vertebræ into position, is not a very pleasing one, especially to those who understand what may be happening inside.

In estimating the value of this proceeding, and its place in surgery, we must take into consideration on the one hand the facts placed at our disposal in the papers read by Mr. Tubby and Mr. Jones, and those given by M. Calot and Mr. Murray in the discussion which followed, and on the other our general surgical experience.

Now in regard to what we were told at this meeting it has to be observed that, although many successes were related, we heard also of a few failures—of abscess, paralysis, wide diffusion of tuberculosis, and even death ensuing—and we cannot help saying that there seems to be but little clue as to the sort of case in which the one result on the other is likely to occur. Truly we were given various instances in which such accidents had taken place where the treatment had not been applied; it was pointed out that had reduction been practised in these cases it would have been wrongly credited with the result; and *per contra*, it was argued that where such accidents did occur after the treatment they also might have been quite independent of what had been done. This argument, however, we think ought not to be applicable to such cases. No one can deny that this mode of treatment is still on its trial, and that in each case in which it is practised it is a matter of serious experiment, and we cannot but think that patients on whom such tentative methods are to be tried should be placed amid such conditions, and kept so long under observation beforehand, as to eliminate, in a very great degree, this source of error.

Again, it is worthy of note that in some of the cases the spines could not be straightened, notwithstanding all the force which was applied; that in others the operation had to be repeated several times, while in some of the apparent successes the straightening was not permanent.

We are forced, then, to fall back on general principles, to consider not results only, but processes, and to ask what happens when an angular curvature of the spine, resulting from tuberculous disease, is forcibly straightened? As to this, we are given but meagre information. A moment's consideration of the mechanics of the problem, however, is enough to show that the answer does not depend entirely, or even mainly, on the condition of the originally-diseased bodies of the vertebræ and intravertebral cartilages. It is clear that, so far as these parts are concerned, very considerable deformity can be rectified, and a very considerable amount of adventitious supporting material can be easily torn through. The difficulty lies in the posterior half of the spinal column; and, although this point does not seem to have received much consideration by the authors of the papers, the remarks made by M. Calot showed how real may be the obstruction caused by adhesions or bony growths between the spinous processes and laminæ, for if these become fixed in their mal-position, it may be quite impossible to secure replacement. Hence, M. Calot's suggestion that the offending bony plates should be divided, or even the spinous processes removed by a preliminary operation. Such a proceeding as this, however, takes away much from the apparent simplicity of the method, and puts it in quite a fresh category. Analogy with other instances of tuberculous disease has been much invoked to prove the justifiability of forcible straightening of the spine. If the knee, it has been asked, why not the spine? It must be pointed out, however, that we possess far better means of knowing the condition of the parts involved, and infinitely more power of regulating the force employed, in regard to the knee than the spine; and, for all that, we do not think that most surgeons are much in love with forcible straightening after tuberculous disease, even in the knee, if the displacement has gone far.

Finally, we come to the results. Is the gap filled up by bone? According to reported skiagraphic examinations, this would seem to be the case; but we should like to see a few post-mortems. Nothing is more interesting than the power and strength which many patients, who have suffered from Potts' disease in early life, ultimately develop, notwithstanding their deformity; and it must be remembered that this is a disease which is common among the poor, to whom, as Mr. Murray put it, a strong, though crooked, back is more desirable than a straight, but "wobbly," one. It is far too early as yet to say whether the new process will give such good results in regard to strength, for most even of M. Calot's cases are still wearing jackets.

The matter will come up at another meeting of the Clinical Society for that further consideration which it well deserves. The method is a most interesting and important one, but, before it meets with general approval, we ought to know much more than we do as to the choice of cases and as to the ultimate results. In the meantime, it may be well to consider whether it does not teach that we ought to take far more pains than is at all usual to prevent the occurrence of distortion during the whole progress of the disease. The old idea that the production of angular curvature is but a step in the cure of the disease may require some modification.

